# Microencapsulation to Harness the Antimicrobial Potential of Essential Oils and Their Applicability in Dairy Products: A Comprehensive Review of the Literature

**DOI:** 10.3390/foods13142197

**Published:** 2024-07-11

**Authors:** Handray Fernandes de Souza, Fabio Ribeiro dos Santos, Jeferson Silva Cunha, Flaviana Coelho Pacheco, Ana Flávia Coelho Pacheco, Maria Eduarda Marques Soutelino, Caio Cesar Nemer Martins, Irene Andressa, Ramon da Silva Rocha, Adriano Gomes da Cruz, Paulo Henrique Costa Paiva, Igor Viana Brandi, Eliana Setsuko Kamimura

**Affiliations:** 1 Department of Food Engineering, School of Animal Science and Food Engineering, University of São Paulo, Av. Duque de Caxias Norte, 225, Pirassununga 13635-900, SP, Brazil; handrayfds@usp.br (H.F.d.S.); ramonrocha@usp.br (R.d.S.R.); 2 Department of Food Technology, Federal University of Viçosa, University Campus, Viçosa 36570-900, MG, Brazil; fabio.r.santos@ufv.br (F.R.d.S.); jeferson.cunha@ufv.br (J.S.C.); flaviana.pacheco@ufv.br (F.C.P.); irene.andressa@ufv.br (I.A.); 3 Instituto de Laticínios Cândido Tostes, Empresa de Pesquisa Agropecuária de Minas Gerais (EPAMIG), Lieutenant Luiz de Freitas, 116, Juiz de Fora 36045-560, MG, Brazil; ana.f.pacheco@ufv.br (A.F.C.P.); paulohcp@epamig.br (P.H.C.P.); 4 Department of Food Technology, College of Veterinary, Fluminense Federal University, Niterói 24230-340, RJ, Brazil; mariaems@id.uff.br; 5 Forest Engineering Department, Federal University of Viçosa, University Campus, Viçosa 36570-900, MG, Brazil; caio.martins@ufv.br; 6 Department of Food, Federal Institute of Science and Technology of Rio de Janeiro, Rio de Janeiro 20270-021, RJ, Brazil; adriano.cruz@ifrj.edu.br; 7 Institute of Agricultural Sciences, Federal University of Minas Gerais, Av. Universitária, 1000, Montes Claros 39404-547, MG, Brazil; ibrandi@ica.ufmg.br

**Keywords:** food, natural preservatives, dairy industry

## Abstract

This literature review explores cutting-edge microencapsulation techniques designed to enhance the antimicrobial efficacy of essential oils in dairy products. As consumer demand for natural preservatives rises, understanding the latest advancements in microencapsulation becomes crucial for improving the shelf life and safety of these products. The bibliometric analysis utilized in this review highlighted a large number of documents published on this topic in relation to the following keywords: essential oils, AND antimicrobials, AND dairy products, OR microencapsulation. The documents published in the last 11 years, between 2013 and 2023, showed a diversity of authors and countries researching this topic and the keywords commonly used. However, in the literature consulted, no study was identified that was based on bibliometric analysis and that critically evaluated the microencapsulation of essential oils and their antimicrobial potential in dairy products. This review synthesizes findings from diverse studies, shedding light on the various encapsulation methods employed and their impact on preserving the quality of dairy goods. Additionally, it discusses the potential applications and challenges associated with implementation in the dairy industry. This comprehensive analysis aims to provide valuable insights for researchers, food scientists, and industry professionals seeking to optimize the use of essential oils with antimicrobial properties in dairy formulations.

## 1. Introduction

Milk and dairy products have been important elements of the human diet since ancient times and have great nutritional value [1]. As they are rich in proteins, fats, carbohydrates, and vitamins, milk and dairy products are an ideal medium for developing and propagating pathogenic and non-pathogenic microorganisms [2,3]. Therefore, they are sold in vacuum or modified atmosphere packaging, mostly stored under refrigeration or freezing conditions, such as pasteurized milk, cheese, butter, yogurt, and ice cream [2]. On the other hand, under unhygienic conditions, the growth of pathogenic and non-pathogenic microorganisms in milk and dairy products stored for several days causes serious concerns regarding the quality, shelf life of the product, and consumer health [4]. For this reason, there is growing interest in applying essential oils that have antimicrobial properties directly to milk and dairy products or incorporating them into packaging materials.

Essential oils (EOs) are hydrophobic aromatic compounds that have demonstrated antimicrobial action against several microorganisms in dairy products such as milk [5,6,7], cheese [8,9], yogurt [10,11], ice cream [12,13], cream [14], and butter [15]. Due to their hydrophobic nature, EOs easily penetrate the bacterial cell wall, interfering with the molecule transport mechanism and causing cell inactivation. On the other hand, EOs are subject to degradation under certain environmental and processing conditions, such as light, high temperature, humidity, and extreme pH [16]. Thus, encapsulation is a method used to protect these compounds so that they can confer excellent antimicrobial properties [17].

Encapsulation is the process of coating small particles of solid, liquid, or gaseous components with a protective coating material [16]. Despite several advantages, studies on the encapsulation of EOs and application in milk and dairy products are rare [18,19], with few studies evaluating antimicrobial properties [1,3,20,21]. Given this context, this review study presents an interesting novelty that consists of gathering scientific evidence on the advances of microencapsulation to harness the antimicrobial potential of EOs and applications in dairy products, considering the scientific literature of the last 11 years, in the period between 2013 and 2023. Therefore, this article aims to present a comprehensive review of the encapsulation of EOs and antimicrobial potential in milk and dairy products. In parallel, a bibliometric analysis was used to elucidate trends and perspectives, as well as gaps in this area of research. The analysis focused on review articles and research published between 2013 and 2023.

## 2. Bibliographic Research Methodology

For bibliographic research, the document search methodology adopted by Souza et al. [22] from the Scopus database (https://www.scopus.com/search/form.uri?zone=TopNavBar&origin=searchadvanced&display=basic#basic, accessed on 18 April 2024). The following document search filter was used: “Search within: Article title, Abstract, Keywords” and “Search documents”. The document search used four keywords, described below: essential oils, AND antimicrobials, AND dairy products, OR microencapsulation.

A time interval of the last 11 years was considered when searching for the documents, with information collected between 2013 and 2023. Then, documents were filtered to consider only the “Article” and “Review” types. All information acquired in the document search was exported in CSV (Excel) format. Afterward, a bibliometric analysis was carried out using the VOSviewer software version 1.6.19 (https://www.vosviewer.com/, accessed on 18 April 2024) [23], according to the methodology described by Teixeira and Ferreira [24] and also adopted by Souza et al. [22]. In addition, to obtain a more robust and highly relevant comprehensive literature review, important scientific articles from other databases such as Web of Science, Science Direct, Scielo, and Google Scholar were also used.

## 3. Bibliometric Analysis of the Last 11 Years: Period between 2013 and 2023

**Figure 1:**
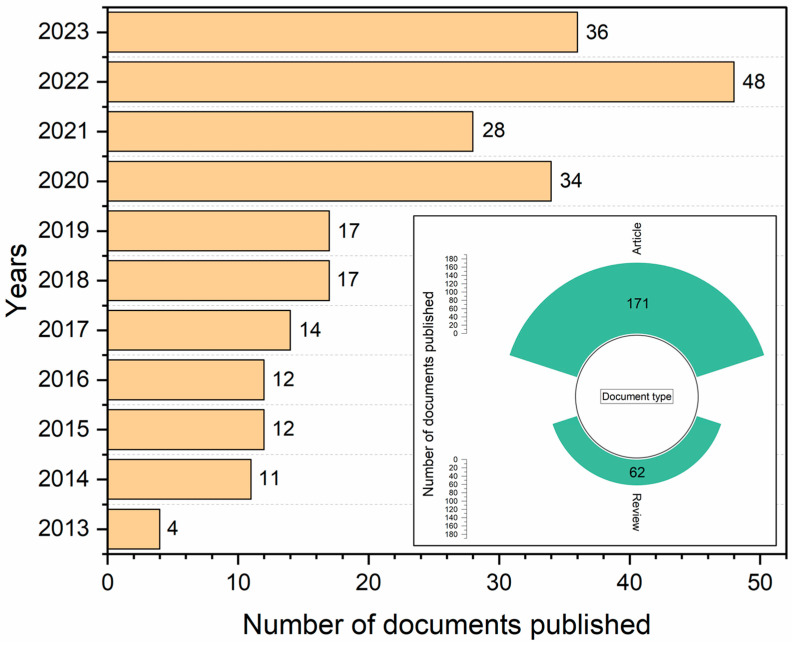
Number and type of documents published between 2013 and 2023 in the Scopus database.

Bibliometric analysis offers an interesting interpretation, as it allows us to evaluate the data and scientific knowledge available in the literature, objectively analyze a subject, and allows the description of researchers, journals, countries of origin, occurrence of keywords, and funding institutions [24,25,26,27], in addition to enabling us to analyze the challenges, advances, and perspectives of scientific research [22,26]. According to Scopus, 233 documents published between 2013 and 2023 were identified, as shown in Figure 1. It is observed that most documents were published in the last six years, between 2018 and 2023. Regarding the types of documents published, 171 are research articles, corresponding to 73.39% of the total publications, and 62 are reviews, corresponding to 26.61% of publications (Figure 1).

**Figure 2:**
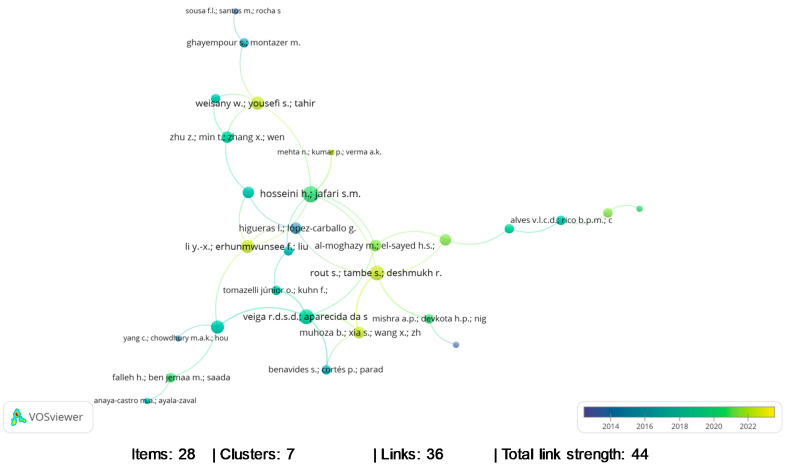
Bibliographic coupling in relation to the authors of most cited documents, considering the bibliometric analysis between the years 2013 to 2023, in the Scopus database.

The bibliometric analysis showed a network mapping in bibliographic coupling relating to the authors of the most cited documents (Figure 2). It can be seen in Figure 2 that the trend towards the yellow color highlights authors with recent publications for the year 2023, while the trend towards the blue color highlights authors with older publications for mid-2013. The 28 most cited authors are grouped into seven large clusters, and within each cluster, the authors are associated with documents published in the same database. Furthermore, of the 233 documents published between 2013 and 2023, the 10 most cited authors are Galié et al. [28] (561 citations), Falleh et al. [29] (333 citations), Yang et al. [30] (219 citations), Omonijo et al. [31] (186 citations), El-Sayed et al. [32] (169 citations), Tao et al. [33] (148 citations), Mahato et al. [34] (141 citations), Dima et al. [35] (139 citations), Bora et al. [36] (138 citations), and Zanetti et al. [37] (131 citations).

**Figure 3:**
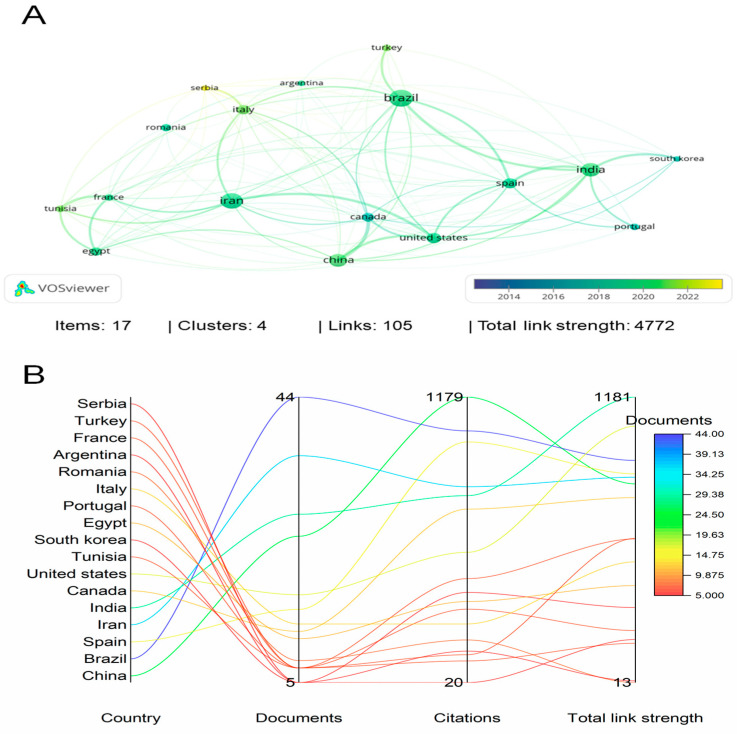
Bibliographic coupling in relation to the countries with the most published documents (**A**) and citations (**B**), considering the bibliometric analysis between the years 2013 to 2023, in the Scopus database.

Figure 3 presents a network map in bibliographic coupling relating to the countries with the most published documents. Figure 3A shows that the tendency towards yellow stands out in countries with recent publications, such as Serbia and Tunisia, and the tendency towards blue stands out in countries with old publications, such as Portugal, Canada, and South Korea. Figure 3B presents a list of countries with the number of published documents and citations. Among the 17 countries, Brazil has the largest number of published documents (44 documents), followed by Iran (36 documents), India (28 documents), and China (25 documents). However, regarding the number of citations, China ranks first with 1179 citations, followed by Brazil, Spain, Iran, India, and Canada with 1042, 997, 815, 779, and 724 citations, respectively. Interestingly, Brazil, Iran, and China remained among the top four regarding the number of documents and citations.

**Figure 4:**
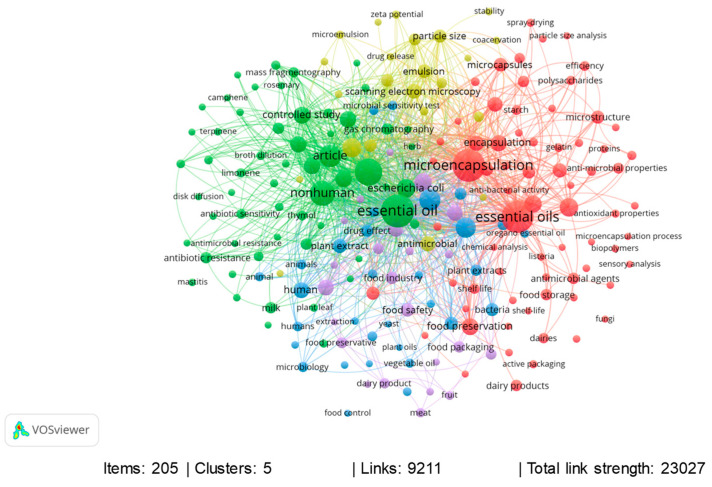
Co-occurrence for all keywords, considering the bibliometric analysis between the years 2013 and 2023, in the Scopus database.

The co-occurrence analysis performed for all keywords shows a network map with the most used keywords in published documents (Figure 4). Around 205 keywords were identified and grouped into five large clusters. Figure 4 presents each network of keywords that generally have co-occurrence in the same databases in different colors. In general, the keywords in the co-occurrence map (Figure 4) are strongly related to essential oils and their antimicrobial action, with an emphasis on different types of microorganisms such as *Escherichia coli*, *Staphylococcus aureus*, *Listeria monocytogenes*, and *Bacillus cereus*, microencapsulation, microencapsulation mechanisms, food preservation, and bioactive compounds present in EOs. In this sense, the five most frequently occurring keywords, according to Figure 4, are essential oil (110 occurrences), microencapsulation (105 occurrences), essential oils (104 occurrences), antimicrobial activity (73 occurrences), and nonhuman (63 occurrences). Based on the approaches and information obtained through a bibliometric analysis, it can be highlighted that a large number of published documents and scientific knowledge are available on this topic in relation to the following keywords: essential oils, AND antimicrobials, AND dairy products, OR microencapsulation. The documents published in the last 11 years, between 2013 and 2023, show a diversity of authors and countries researching this topic and the keywords commonly used. However, no study was identified in the literature consulted that brought together this available information and critically evaluated the advances in microencapsulation of essential oils and their antimicrobial potential in dairy products. Therefore, this study, based on a bibliometric analysis and a comprehensive review of the literature, is fundamentally important given the relevance of this subject.

## 4. Essential Oils (EOs) and Their Antimicrobial Action

Essential oils (EOs), also known as ethereal oils, are volatile secondary metabolites formed by bioactive compounds of a complex nature and low molecular weight, found in plants or in the parts of plants such as leaves, stems, seeds, flowers, fruits, and roots [38,39]. These substances have intrinsic properties such as natural origin, volatility, and complexity in their composition and are liquid substances with a prominent aroma [40,41]. In plants, these substances represent a small part of their composition, with approximately 5% of the plant’s dry matter, so they are directly involved in their defense mechanisms or even in attracting insects to favor the dispersion of pollen and seeds [42,43].

Regarding the composition of EOs, they are generally formed by lipophilic compounds, and in rare cases, they are soluble in water when associated with a solvent. They can be divided into two main groups: hydrocarbons (terpenes and sesquiterpenes) and oxygenated compounds (acids, alcohols, aldehydes, acetals, ketones, oxides, phenols, ethers, esters, and lactones) [44,45]. Such a composition allows the odor and flavor characteristics of each EOs to be defined. These substances have been widely used for their properties already observed in nature, i.e., their antifungal, antibacterial, and insecticidal activities [29,46,47].

Around 3000 EOs are known, among which approximately 300 have been tested and recommended in a range of products, such as preservatives and flavorings in foods, cosmetics, insecticides, herbicides, pharmaceuticals, and fragrances [38,48,49]. This literature review focuses, in particular, on EOs with antimicrobial properties. Therefore, Table 1 presents the main plants producing EOs with antimicrobial action.

Studies have been carried out to evaluate the application of essential oils as antimicrobial agents in foods. Coriander, oregano, and thyme oils have been used to control pathogens and preserve the quality of beef and chicken [80,81,82]. Oregano oil has been used to extend the shelf life of fish and seafood [83,84]. Another application included the edible coating of apples using lemongrass and oregano oils, increasing their durability [85]. In another study, nanoencapsulated cinnamon, palmarosa and lemon balm oils were used with the aim of extending the shelf life of minimally processed melon [86]. It is clear that essential oils are promising and suggest that they could replace chemical additives and compose more natural and safe food products, with a long shelf life. However, research is still in its early stages and requires new validation tests so that they can be used on an industrial scale.

Secretory glands are the structures responsible for storing EOs in plants. Therefore, the extraction process begins with drying the plant material in the shade for a week and, shortly after, the EOs and extracts containing volatile components are extracted from these structures using different extraction methods [87]. These extraction methods can be grouped into traditional methods, such as hydrodistillation [88] and steam distillation [89], and innovative methods, such as ultrasound-assisted extraction [90], supercritical fluid extraction [43], and microwave-assisted extraction [91]. It is important to highlight that the choice of extraction method is influenced by the portion of the plant material (flower, leaf, stem, root or seed), its physical form, in addition to the economic viability of the process. Inappropriate choice of extraction method can alter the chemical composition of the EOs, resulting in the loss of bioactivity [92,93].

After identifying, quantifying, and extracting the EOs, it is important to study the antimicrobial activity of each compound present, allowing us to evaluate whether there is interaction with the other constituents of the EOs and how its mechanism of action occurs [42]. In view of this, the antimicrobial activity of EOs has already been evaluated by various mechanisms reported in the literature and which describe the action of essential oils on bacterial and fungal cells [94,95].

**Figure 5:**
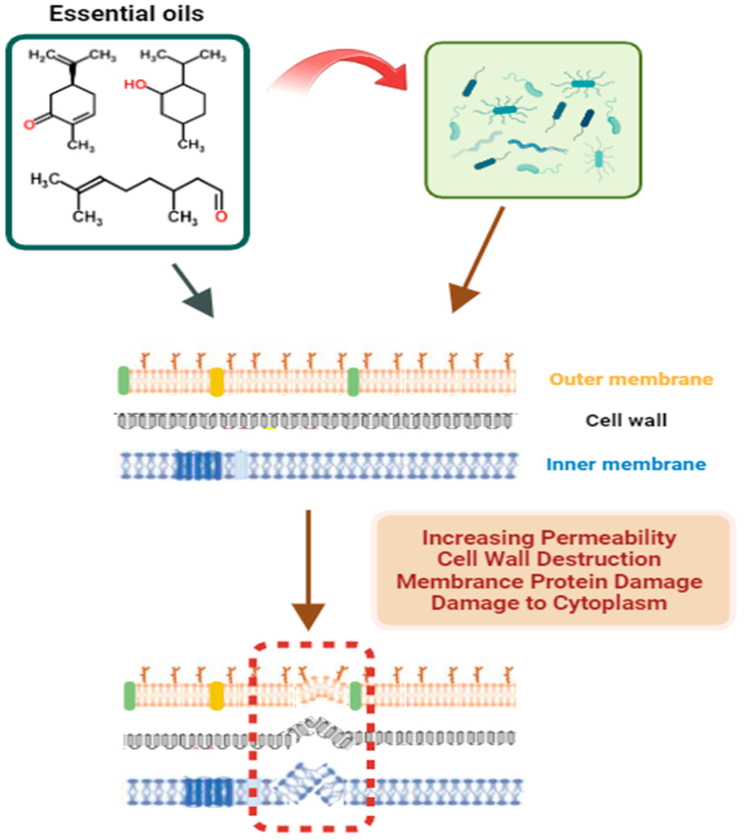
Mechanism of action of essential oils (EOs) on bacteria.

The best known mechanism of action is the change in the permeability of the cell membrane of microorganisms through the interaction of lipophilic compounds present in EOs (Figure 5). A phospholipid bilayer forms the cytoplasmic membrane, and the actions caused by its integrity result in changes in the functioning of the electron transport chain, influencing the absorption of nutrients, as well as modifications in the synthesis of proteins and nucleic acids, changes in the coagulation of cell content, and the inhibition of various enzymes that are essential for the metabolism of the microbial cell [96]. In this sense, in general, microorganisms such as Gram-positive bacteria tend to be more susceptible to the antimicrobial activity of EOs when compared, for example, with Gram-negative bacteria due to differences in the composition of the cell wall [97,98]. 

The cellular structure of Gram-negative bacteria is complex, consisting of a thin peptidoglycan layer and covered by an external membrane formed by hydrophilic lipopolysaccharide, functioning as a permeable selective barrier. This outer membrane plays a role in limiting the diffusion of hydrophobic compounds from EOs, thus preventing bioactive compounds from accumulating in the cell membrane [99,100]. With regard to the composition of the cell wall of Gram-positive bacteria, it is generally composed of approximately 90 to 95% peptidoglycan. This also allows EOs to infiltrate and act on the cytoplasmic membrane, increasing their antimicrobial action and efficiency [101]. According to Lopes-Romero et al. [102], cell shape is another aspect that can contribute to the susceptibility of bacteria, so rod-shaped bacteria are more susceptible than cocci-shaped bacteria.

Fungi are generally more resistant to antimicrobial action than bacteria since many species present intrinsic resistance to most available antifungals [103]. According to Mani-López et al. [104], the antifungal action of EOs occurs through the rupture of the cell wall, altering cell membranes, coagulating the cytoplasm, and damaging cell organelles, resulting in the leakage of cell contents. Another method of action is proposed by Alderees et al. [105], who report that the lysis of the fungal cell membrane is generated by interactions that unite EOs with ergosterol, which is the main sterol present in fungal cell membranes, and is responsible for controlling permeability and fluidity.

The literature reports numerous studies demonstrating the advantages and possibilities of using EOs. However, using EOs in low-fat foods, resulting from their hydrophobic nature, presents a challenge for their application. Furthermore, the intense aroma and flavor of EOs limit their application in high doses in certain food products due to possible undesirable sensory characteristics. Therefore, it is fundamental and necessary that encapsulation techniques are applied to EOs to improve their application and expand their industrial use.

## 5. Microencapsulation of Essential Oils (EOs) with Antimicrobial Potential

EOs have important antimicrobial properties. However, their direct application to food is restricted due to their strong aroma and characteristic flavor that can cause undesirable sensory changes, in addition to their low stability when exposed to light, oxygen, and temperatures above 26 °C. One of the biggest obstacles related to using EOs is their low solubility in the aqueous phase of food formulations [106]. As a result, several studies have pointed to encapsulation as a viable and effective technique to increase the protection of these compounds against external factors and, consequently, reduce the impact on sensory characteristics and increase dispersibility in areas of food where microorganisms can develop [107,108].

Encapsulation is when liquid, solid, or gaseous agents are adsorbed, or entrapped through a coating. The encapsulated component, called active, generally constitutes the core(s) of the capsule, while the material that surrounds or adsorbs the active is known as wall material or carrier agent [109]. Wall materials commonly used for the encapsulation process of food products must achieve high encapsulation efficiency and homogeneous distribution in capsules, have high loading capacity, and possess the required controlled release characteristics [110].

Regarding the encapsulation of lipophilic functional food ingredients such as EOs, the main wall materials used include polysaccharides (starch and cellulose), phospholipids and proteins (albumin and casein), or the association of two or more of these materials [111]. Among the available encapsulation techniques, the two commonly used methods for EOs are microencapsulation and nanoencapsulation. The occurrence of microencapsulation is classified into three main categories: (i) chemical processes (e.g., interfacial polymerization), (ii) physico-chemical processes (e.g., complex coacervation), and (iii) physical processes (e.g., spray drying) [112]. EO capsules can vary in size depending on the wall materials and the encapsulation technique [113]. Table 2 presents, in general, the main techniques used to encapsulate EOs with antimicrobial potential.

The encapsulation of bioactive compounds, such as EOs, can improve stability (physical, chemical, and thermal), biological activity, and solubility in gastrointestinal fluids while also enabling controlled release and targeted delivery [25,134,135]. It is important to note that the lipid digestion of oils under isolated conditions differs when there are non-lipid components, such as polysaccharides, proteins, and fibers (wall materials). In one study, it was pointed out that these non-lipid components have a very high influence on the degree and kinetics of lipid digestion, as well as on bioavailability [136].

Understanding the mechanisms of lipid digestion and the factors that affect its degree and kinetics will allow an appropriate choice of encapsulation materials, so that the release and physiological digestion of lipids can be controlled according to the body’s needs, eventually reducing the risk of disease resulting from a greater intake of oils [137]. For example, Timilsena et al. [138] and Timilsena et al. [139] reported that the pattern and kinetics of the digestion of non-encapsulated oils show differences when compared to the digestion of encapsulated oil. These authors showed that the digestion of encapsulated oil was affected by the nature of the wall materials in which these oils were encapsulated. Protein-based capsules are digested in the stomach and release the oil from the matrix. On the other hand, gum-based capsules are not digestible but are solubilized in the stomach and intestine. In addition, the protein–gum complex capsules were more resistant to digestion and resulted in less oil being released in the gastric phase and more intact oil reaching the intestinal phase. These authors also pointed out that the slower digestion of encapsulated oil occurs because the oils need to be released from the matrix before being affected by the enzyme (lipase) [138,139]. Furthermore, these wall materials affect the interfacial behavior and interfere with the diffusion and adsorption of lipase into the oil droplets [138,139].

One of the advantages of microencapsulation techniques is the controlled, targeted release of the active agent, which is based on mechanisms of diffusion or rupture of the microcapsule wall [140]. An encapsulation method must be fast, easy, reproducible, and readily adaptable for industrial scale-up. Microencapsulation methods present different approaches (Table 2) whose advantages, limitations, and appropriate applications must be evaluated simultaneously and systematically in order to choose the ideal method for the intended purpose. The main techniques are briefly described.

### 5.1. Microencapsulation Techniques

#### 5.1.1. Interfacial Polymerization

Interfacial polymerization is a process that involves the reaction of two types of monomers or a monomer and a catalyst dissolved in two immiscible phases [141]. It is being explored in several areas, from the incorporation of medicines to the encapsulation of peptides and essential oils [142]. In this technique, to optimize membrane quality and yield, it is important to consider process variables, such as monomer concentrations, reaction duration, and mixing intensity [16].

Among its positive aspects, its simplicity, reliability, low cost, control of the average capsule size and membrane thickness, and high encapsulation efficiency stand out [16,143]. However, its use is hampered by the need for a large amount of (non-biodegradable) solvents and the challenging control of the polymerization reaction [143,144].

#### 5.1.2. Complex Coacervation

In the technique known as complex coacervation, the oil phase is formed by a combination of two or more polymers whose opposite charges result in phase separation when the pH is adjusted [145]. The microcapsules produced by this method can have an average size between 1 and 1000 μm [16].

One of the great advantages of complex coacervation is associated with the content and stability of the oil inside the capsule, resulting in a lower concentration of superficial oil [16,134]. Complex coacervation, while offering simplicity, scalability, affordability, reproducibility, and solvent-free benefits over other microencapsulation techniques, does have its limitations. It is sensitive to even minor pH variations, which could restrict its use [146].

#### 5.1.3. Ionic Gelation

Ionic gelation is based on the ionic crosslinking of a polymer in the presence of multivalent cations (Ca^2+^, Ba^2+^ and Al^3+^) and is used to encapsulate bioactive compounds, medicines, or probiotics [147]. This technique shares characteristics similar to complex coacervation in terms of simple production control, low cost, and high encapsulation efficiency. However, its disadvantages include dispersion heterogeneity, polydispersity, and the need for controlled agitation [148].

#### 5.1.4. Spray Drying

In the food industry, spray drying is the predominant method of encapsulation. This method promotes the drying of the material by pumping it into the drying chamber of the spray dryer. The result of this is a fine powder (10–50 µm) and larger particles (2–3 mm) free of moisture and low water activity, which helps to control the risks of microbial contamination of products [134].

Spray drying, while widely used in the microencapsulation of essential oils due to its efficiency and equipment availability, is not without its challenges. The scarcity of good wall materials and the agglomeration properties of microcapsules in fine powder (which negatively impact stability and solubility) pose significant limitations for their diffusion [123,134,146,149].

### 5.2. Nanoencapsulation Techniques

The various nanoencapsulation methods, including those mentioned in Table 2, are considered relatively simple [150,151,152]. Among the methods, the β-cyclodextrin molecular inclusion technique deserves to be highlighted. It is recognized as an effective strategy for improving the bioavailability, stability, and solubility of compounds that are poorly soluble in water. However, its cost and selectivity restrict its application [150].

Although the basic principle of encapsulation is similar for all methods, it is important to note that the choice of the appropriate technique can depend on different factors, such as the size of the particles, the chemical and physical properties of the core as well as the wall, the application of the microencapsulated material, the types of controlled release mechanisms, and even the costs [153].

## 6. Application in the Dairy Industry

The application of EOs in the food industry has been gaining ground and standing out in food preservation. This fact is related to the high antimicrobial activity of EOs, given the potential for use by the food industry to improve the quality of food, thus increasing its shelf life and reducing the growth of pathogenic bacteria [2,107,154,155,156]. 

Among the known EOs, only a small portion are of commercial interest for applications in the food industry [43,155], as their effectiveness varies depending on the form of application, the concentration used, the storage temperature, and the food matrix and sensory characteristics of food [2]. Encapsulation, as mentioned previously, has become a great alternative, as it alters the physical properties of the compounds, providing control and targeted release of the active ingredients [25]. Thus, several studies have presented investigations on the application of different microencapsulated EOs and their antimicrobial action in food systems, for example, in dairy products, as shown in Table 3.

### 6.1. Milk

Due to the chemical composition and high water activity in milk, this food presents an excellent medium for the growth and multiplication of spoilage and pathogenic microorganisms [165]. Therefore, safety, mainly relating to its bacteriological quality, is a major concern for dairy industries and public health communities [166]. Although the pasteurization process is quite consolidated, the total destruction of microorganisms does not occur, but rather their inactivation, thus allowing them to grow slowly or produce spores [167]. To avoid post-pasteurization contamination, new approaches have been adopted in food preservation, including the addition of natural compounds with inhibitory or bactericidal activities, for example, essential oils (EOs).

When evaluating the antimicrobial effects in milk, examining the interference and potential cross-reactions of the antimicrobial with food constituents, such as proteins and fats, is crucial. These interactions can be challenging to overcome and often require substantial amounts of antimicrobials to significantly reduce a product’s pathogen load. Several natural compounds with antimicrobial action have already been encapsulated and applied to dairy products, and have shown positive results in improving the quality and microbiological safety of these products, for example, in milk [155]. Locali-Pereira et al. [158] observed that free pink pepper oil showed no inhibitory effect on *S. aureus* and *L. monocytogenes* bacteria in both whole and skim milk, suggesting that its bioavailability may have been reduced due to interactions with other milk components. However, oil microencapsulation resulted in bacterial inhibition, especially in skim milk, indicating that interactions between the oil and milk lipids may have affected its efficacy as a natural preservative. Double-layer microencapsulation was even more effective in inhibiting bacterial growth in both types of milk, due to the greater retention of volatile compounds in this structure, suggesting that the additional coating may act as an extra barrier against interactions of encapsulated materials with components of the food matrix. In line with these findings, Shah et al. [20] revealed that complete inhibition was observed in all treatments with 6.5 g/L of eugenol in skim milk, demonstrating the compound’s efficacy. Furthermore, nanodispersion exhibited superior antimicrobial activity to free eugenol, with only 3.5 g/L of eugenol needed to achieve inhibition. However, as fat levels increased in whole milk, an increase in eugenol concentration was required to inhibit the growth of *E. coli* O157:H7. This trend of improvement in antimicrobial activity with nanodispersion was also observed against the Lm Scott A strain, although to a lesser extent than the results against *E. coli*.

EOs have been combined with known antimicrobials, such as nisin, to increase antimicrobial activity, especially targeting effects against Gram-negative bacteria. Nisin is a bacteriocin effective against a variety of Gram-positive pathogens, including *Bacillus* and *Clostridium*, and can inhibit the growth of their spores [21]. However, its use as a food biopreservative is limited by the lack of effect against Gram-negative bacteria. Pinilla and Brandelli [21] observed that free and liposomal nisin–garlic extract resulted in a difference of 1–4 log CFU/mL for the tested strains when compared with free nisin and garlic extract separately. Compared to the control, a difference of 5–6 log CFU/mL in viable counts of Gram-positive strains and 3–4 log CFU/mL for Gram-negative bacteria was observed for nisin–garlic extract treatments.

### 6.2. Cheese

Cheese, one of the most globally consumed dairy products, faces significant challenges from contamination by deteriorating microorganisms. These microorganisms reduce cheese’s shelf life and pose risks to consumer health. While chemical preservatives can mitigate this issue, there is a growing demand for healthier, preservative-free foods. This has spurred the search for natural preservatives that can ensure food safety and prolong cheese’s shelf life [168].

Some studies highlight the potential of EOs as natural and sustainable preservatives, with notable antimicrobial activity against deteriorating microorganisms found in cheese. Fernandes et al. [162] observed that microencapsulated rosemary oil effectively controlled bacterial growth, particularly evident after 3 days of storage, with reductions of 1.36 log cycles in mesophilic bacterial counts. Moreover, higher counts were observed after 3 days of storage for the control treatment, 8 days for bulk essential oil, and 12 days for microencapsulated essential oil. This prolonged effect in the microencapsulated essential oil treatment may be attributed to the gradual release of active constituents, allowing for an extended antimicrobial action. Regarding coliforms, their development in the cheese was unaffected by any treatment, likely due to their Gram-negative nature, which has been noted to be less susceptible to the effects of essential oils compared to Gram-positive bacilli, possibly due to their lipopolysaccharide composition, which avoids the components to enter the cytoplasmic membrane. In another study, Melo et al. [159] observed that microencapsulated lemongrass essential oil controlled the proliferation of microorganisms over 21 days of storage, showing more satisfactory results than non-microencapsulated lemongrass oil.

Studies have also been conducted investigating different encapsulation methods, such as microencapsulation or nanoemulsification. Pérez-Soto et al. [3] observed that microencapsulated and nanoemulsified orange essential oil significantly reduced mesophilic aerobic bacteria, molds, yeasts, and coliforms compared to free oil, with nanoemulsification proving more effective. The authors attributed this effect to the synergy between the bioactive compounds of the nanoemulsion. Additionally, although particle size in nanoemulsions may contribute to membrane penetration, the authors believe that this does not necessarily correlate with increased functionality, as the antimicrobial activity of nanoemulsions is attributed to the entire system.

Other crucial characteristics are the concentration of EOs in the formulation, the immersion period of cheese slices in nanoemulsions, and the storage temperature, which can significantly influence their bactericidal activity. Artiga-Artigas, Acevedo-Fani, and Martín-Belloso [163] demonstrated that oregano essential oil and tangerine fiber reduced the microbial population by 1.4 and 1.5 log CFU/g, respectively, in cheese pieces coated with 2.0% or 2.5% *w*/*w* EOs over 15 days of refrigerated storage. However, coatings with 1.5% *w*/*w* EOs were ineffective in reducing the population of *S. aureus*. Additionally, coatings with 2.0% *w*/*w* effectively delayed the growth of psychrophilic bacteria, molds, and yeasts. In the case of coatings prepared with 2.5% *w*/*w* EOs, molds and yeasts were not grown for at least 24 days of experimentation. In a study, Bedoya-Serna et al. [161] found that nanoemulsified oregano essential oil exhibited more notable antifungal activity when cheese slices were kept at 25 °C after 30 min of immersion. The nanoemulsions effectively inhibited the growth of *Cladosporium* sp. and *Fusarium* sp. for up to 30 days, while for *Penicillium* sp., only the highest concentrations of the nanoemulsion were able to inhibit growth until the seventh day. This suggests a greater resistance of this fungus to the antifungal effect of nanoemulsified oregano oil.

Recently, Sassi et al. [169] evaluated the encapsulation of an antifungal formulation of citrus extract and EOs rich in cinnamaldehyde, followed by lecithin/SMP with HBL10, and were applied for the preservation of grated cheese. Among the main results obtained in this study, it was observed that in the in situ microbiological tests, the application of the formulation effectively inhibited the growth of yeasts and molds for up to 70 and 77 days in grated cheese stored at 4 °C in sealed packages under air (AS) and under nitrogen (SL), respectively.

### 6.3. Yogurt

Yogurt is among the most popular dairy products, with great nutritional value due to its high protein content [170,171]. Like milk, yogurt can present challenges related to microbial quality. In this context, several studies have also been developed to evaluate the effect of EOs as a natural preservative in yogurt. Furthermore, including EOs in the nanoencapsulated system can improve physical properties such as dispersion, stability, turbidity, viscosity, and microbiological quality, thus contributing to its functional activity compared to free EOs.

A recent study by Haseli et al. [1] explores the potential of encapsulated Mofarrah (*Nepeta crispa*) essential oil as a preservative, taking advantage of its antimicrobial properties. In this study, nanoencapsulated Mofarrah essential oil was used with pectin to extend the shelf life of yogurt since this drink has a high activity of microorganisms. The results demonstrated that the dough sample containing 0.20 mg/mL of nanoencapsulated essential oil had the best inhibitory effect on the population of *E. coli* and *S. aureus* bacteria. Phenolic compounds are mainly responsible for the antimicrobial activity of essential oils, and according to the study, the addition of the oil increased the bioavailability of these compounds, influencing the bacterial population.

Garlic is rich in organosulfur compounds responsible for their antibacterial properties against Gram-negative and Gram-positive bacteria. However, lycine makes it sensitive to high temperatures and pH. Furthermore, Nazari et al. [164] showed that using phytosomes to encapsulate bioactive compounds causes a significant increase in their bioavailability, improving their antioxidant and antimicrobial activities. Therefore, the authors carried out the study using nanophytosomes of garlic essential oil (GEO) as a natural preservative for application in yogurt. As a result, the nanophytosomes loaded with GEO exhibited a greater inhibitory effect against *E. coli* than against *S. aureus*; this difference in the action of GEO is due to the difference in the structure of the cell wall between Gram-positive and Gram-negative bacteria. Gram-positive bacteria have a cell wall comprising a thick layer of peptidoglycan, while Gram-negative bacteria have a double layer of cell membrane. This cell wall difference may be the reason for the better antimicrobial effect of GEO-nanophytosomes against Gram-negative than Gram-positive bacteria.

### 6.4. Other Products

Although studies involving the use of EOs encapsulated in dairy products are limited, their use is not restricted to those mentioned in this study. Other dairy products, such as dairy drinks, kefir, butter, ricotta, and dairy desserts, among others, can be explored for this purpose. For example, recently, Nanakali [172] evaluated the manufacture of nanoencapsulated angelica (*Heracleum persicum*) essential oil in dairy desserts. The encapsulation process resulted in a lower inhibitory effect than pure essential oil due to the presence of a lower percentage of essential oil used in the formulation of these samples. However, the increase in the rate of essential oil in the nanoemulsion formulation increased the inhibitory effect on the bacteria *Escherichia coli* and *Staphylococcus aureus*.

These results demonstrate that the use of EOs and encapsulation techniques present some limitations that may vary according to different factors, such as the type of encapsulation used, the pH of the dairy product, the salt content, and the amount of fat, among others [173].

The pH of dairy products, a key factor in encapsulation, varies depending on the dairy derivative. This variation significantly impacts the stability and durability of the encapsulation, as well as its effect on the food. For instance, yogurt, with its acidic pH of around 4.6, poses a risk of degradation to capsules made from acid-sensitive materials. In such cases, the capsules used in this matrix must be specifically designed to withstand acidic pH levels [164].

The salt and fat content in dairy products can significantly influence the solubility of certain types of encapsulated EOs. A high salt content, for instance, can lead to interaction between the salt and the capsule, potentially compromising stability or accelerating the release of the essential oil. Similarly, dairy products with higher fat content, like butter and cheese, can pose stability challenges, especially if the fat is capable of dissolving the encapsulation matrix [174]. These considerations underscore the importance of careful formulation when dealing with such dairy products.

Furthermore, other factors such as temperature, humidity, exposure to light, and undesirable interactions with dairy matrix constituents can reduce the effectiveness of the encapsulated essential oil [173]. Dairy products are generally subjected to severe heat treatments, such as pasteurization or sterilization. In this case, high temperatures can cause the encapsulation to break, altering the effectiveness of the essential oil. Finally, the milk matrix contains different compounds that can interact with the capsules or EOs, impairing stability or interfering with the controlled release of the contents. Therefore, when considering encapsulation in dairy products, it is essential to evaluate these different characteristics to ensure the performance and safety of the final products [173].

Among other recent applications, a study has formulated and evaluated microparticles of cinnamaldehyde (3-phenyl-2-propenal, E-CIN)—a hydrophobic aromatic aldehyde found in cinnamon bark—and vanillin (4-hydroxy-3-methoxybenzaldehyde, VA), which is a component of vanilla beans, as well as the effect of encapsulation on the inactivation of *Listeria innocua* in a whey protein drink [175]. In this study, it was observed that E-CIN (1.0 g/L) provided greater antimicrobial activity with a reduction of 3.5 log cycles of *L. innocua*, while vanillin encapsulation showed a reduction of 1.0 log cycles of *L. innocua* after 14 days of storage.

## 7. Limitations of This Review Study

This review has brought to light an important and relevant subject for scientific knowledge, highlighting the evidence about microencapsulated EOs with antimicrobial potential for application in the dairy industry. However, it should be noted that this study has some limitations. Among the limitations, it can be highlighted that the high cost of the extraction process and the low yield of EOs offered by some production plants make the industrial process expensive and limited. Other limitations, especially for dairy matrices, include identifying the concentration of the encapsulated agent needed to achieve the desired antimicrobial effect. In addition, the presence of interfering components, particularly fats, strongly influences the activity and ultimately the minimum inhibitory concentration of antimicrobial compounds. Another point that should be noted within the limitations is related to the fact that environmental conditions, such as oxygen in the air, humidity, light, and exposure to high temperatures, can influence EO stability and can degrade EOs, which, when stored improperly, can lead to changes in their chemical composition, and in some situations, can cause a reduction in the antimicrobial potential [176,177]. Therefore, new studies should be carried out and multiple strategies developed to combat these limitations, as well as to favor the application and implementation of microencapsulated EOs in dairy products.

## 8. Conclusions and Perspectives

This review study highlighted the potential of microencapsulating essential oils with antimicrobial action and their application in dairy products. Essential oils are viable and promising alternatives for incorporation into food matrices to extend shelf life and can be used to replace traditional synthetic preservatives. These oils can be obtained from raw materials already widely studied in the literature, such as pepper, cloves, and cinnamon. On the other hand, these oils’ hydrophobicity and strong sensorial characteristics (flavor and aroma, mainly) limit their applicability in the food industry. Thus, microencapsulation is a promising alternative to overcome such limitations and enable the incorporation of these oils into food matrices. Even in well-established heat treatments such as pasteurization, it is still viable to incorporate essential oils as a safety barrier and extend the shelf life of food without compromising its microbiological quality. This aspect highlights the demand for “clean label” food, which is no longer a trend and has become a consumer expectation concerning the product. Another important point is the slogan “Sustainability”, given the use of natural alternatives to chemical and synthetic preservatives for preserving food.

According to the literature, prospects point to applying microencapsulated essential oils with antimicrobial action in dairy products such as milk, cheese, and yogurt. However, more in vitro and in situ research is needed regarding the dosages and behavior of the food matrix when faced with the addition of these compounds throughout the shelf life to provide the consumer with safe foods with adequate sensorial and nutritional quality. Therefore, more studies in this area of research must be carried out.

## Figures and Tables

**Table 1:** Plants producing essential oils (EOs) with antimicrobial capacity.

Common Name	Scientific Name	Compounds with Antimicrobial Action	References
Rosemary	*Rosmarinus officinalis*	α-pinene, camphor, and 1,8 cineole	[50,51]
Garlic	*Allium sativum*	Diallyl disulfide, diallyl trisulfide, and diallyl tetrasulfide	[52,53]
Peppermint	*Mentha × piperita* L.	Menthone, menthol, 1,8-cineole, and limonene	[13]
Angelica	*Angelica archangelica*	E-3-butylidene phthalide, (Z)-ligustilide, (Z)-β-ocimene, and γ-terpinene	[54,55]
Barije	*Ferula gummosa*	α-pinene and β-pinene	[56,57]
Basil	*Ocimum basilicum*	γ-cadinene, α-bergamotene, eugenol, and linalool	[45]
Cinnamon	*Cinnamomum zeylanycum*	Cinnamaldehyde and trans-cinnamaldehyde	[58,59]
Lemon grass	*Cymbopogon citratus*	Neryl acetate, eraniale, geraniol, neral, α-myrcene, linalool, and verbenol	[60,61]
Citronella	*Cymbopogon winterianus*	Citronellol, citronellal, elemol, and linalool	[62,63]
Coriander	*Coriandrum sativum*	Geranyl acetate, camphor, γ-terpinene, linalool, and α-pinene	[64,65]
Clove	*Syzygium* *aromaticum*	α-humulene, α- karyophylene, β-karyophylene, and eugenol	[38,45]
Black cumin	*Nigella sativa*	Nigellicin, thymoquinone, thymol, α-Thujene, p-cymene, and thymohydroquinone	[66,67]
Jamaica pepper	*Pimenta dioica* (L.) Merr.	Eugenol and β-Myrcene	[38]
Lavender	*Lavandula* sp.	Linalyl acetate, camphor, eucalyptol, and linalool	[68,69]
Oregano	*Origanum vulgare*	Carvacrol, p-cymene, gamma-terpinene, and thymol	[70,71]
Allspice	*Pimenta dioica* (L.) Merr.	β-myrcene and eugenol	[72,73]
Pitanga	*Eugenia uniflora*	Germacrene B, Seline-1,3,7(11)-trien-8-one, and seline epoxide-1,3,7(11)-trien-8-one	[74,75]
Sage	*Salvia officinalis*	Camphor, eucalyptol, α-thujone, and borneol	[76]
Wise	*Salvia officinalis*	Borneol, camphor, eucalyptol, and α-thujone	[76,77]
Thyme	*Thymus vulgaris*	Thymol, carvacrol, γ-terpinene, α-pinene, and p-cymene	[78,79]

**Table 2:** Main techniques used to encapsulate essential oils (EOs) with antimicrobial potential.

Encapsulation Technique	Active Material	Wall Materials	Main Results	References
**Microencapsulation**				
Interfacial polymerization	Clove essential oil (*Syzygium aromatum*)	Polyurethane and poly(urea-formaldehyde) (PU and PUF)	- The manufactured microcapsules had great antibacterial activities against the bacteria *Vibrio coralliilyticus*, *Escherichia coli*, and *Exiguobacterium aestuarii.* - Particle size: 102.2 μm. - The microencapsulated essential oils showed a controlled release rate by adjusting the amount of PU reagents and the duration of PUF deposition time.	[114]
	Essential oil of oregano (*Origanum vulgare*) and sage (*Salvia officinalis*)	Polyurea	- Antimicrobial activity against *Penicillium citrinum*, *Rhizopus oryzae*, *Salmonella enterica*, and *Escherichia coli* (>90% reduction in activity). - Particle size: 100 µm (oregano) and 25 µm (sage).	[115]
Complex coacervation	Sichuan pepper essential oil (*Zanthoxylum* L.) (SPEO)	Soluble fiber extracted from Sichuan pepper seeds (SDF) and soy protein isolate (SPI)	- The microcapsules showed good dispersion in water. - High encapsulation efficiency: 91.33%. - Particle size: 6.79 μm. - The antibacterial activity of SPEO was improved after microencapsulation. - Microcapsules provided SPEO with better thermal stability and slow-release property.	[116]
	Rose essential oil (*Rosa damascena*) (REO)	Mung bean protein isolate (MBPI) and apricot peel pectin (APP)	- Encapsulation efficiency of freeze-dried microcapsules: 89.91%. - REO microcapsules showed substantially higher thermal stability compared to free REO. - The coacervate shell of the MBPI-APP complex was also stable in the oral and gastric phases of in vitro digestion. - 65.5% of REO was delivered to the intestinal phase. - Particle size: 10.23 μm.	[117]
	Citronella essential oil (*Cymbopogon citratus*) (CEO)	Gelatin extracted from chrome-tanned leather and sodium alginate	- The best condition was represented by 4% gelatin and 10% CEO, resulting in 83.5% microencapsulation yield. - Encapsulation efficiency: 73.7%. - Particle size: 434.06 μm.	[118]
Ionic gelling	Marjoram essential oil (*Origanum majorana* L.)	Sodium Alginate and Whey Protein Isolate (WPI)	- Encapsulation efficiency ranged from 45.6 to 66%. - Lower concentrations of alginate and WPI resulted in higher encapsulation efficiency. - Particle size: 1.10 to 1.73 μm.	[119]
	Thyme essential oil (*Thymus vulgaris*)	Calcium Alginate	- The best encapsulation conditions were obtained with 2% *v*/*v* of thyme essential oil with a high degree of dispersion (18,000 rpm/5 min). - The microcapsules showed a significant antimicrobial effect, especially on Gram-positive bacteria (*Staphylococcus aureus*). - Particle size: 890 μm.	[120]
	Flaxseed essential oil (*Linum usitatissimum*) (FEO)	Pectin	- The encapsulation efficiency of the oil was 97%. - The oxidative stability of the encapsulated FEO was 13 times greater than that of the oil in its free form. - Particle size: 862 to 1463 μm.	[121]
Spray drying	Cinnamon essential oil (*Cinnamomum zeylanicum*) (CEO)	Whey protein isolate (WPI), maltodextrin (MD), and sodium alginate	- The ideal formulation consisted of 70% wall material (WPI/MD/sodium alginate = 1:3:0.01 (*w*/*w*)) 30% CEO. - The useful life of the microcapsules was 1032 days at 25 °C. - Particle size: 178 to 347 nm.	[122]
	Juniper essential oil (*Juniperus communis* L.)	Gum arabic (GA), maltodextrin (MD), sodium alginate (ALG), and whey protein concentrate (WPC)	- The combination of GA/MD (1:1) as OZ carrier produced microcapsules with the highest encapsulation efficiency (70.07%). - The GA/MD formulation achieved complete and prolonged release of from microcapsules in an oily food matrix. - Particle size: 3.97 to 9.59 μm.	[123]
	Mint essential oil (*Mentha piperita*)	Inulin and gum arabic	- The ideal condition was 35% solid wall, 4% essential oil concentration, and inlet temperature of 110 °C. - The Peppas–Sahlin model was found to be the best approach for SEO launch profiling across four food models. - The optimized spray-dried powder showed faster and greater release in a 50% ethanol medium.	[124]
	Chavir essential oil (*Ferulago angulata)* (*CO*)	Low- and medium-molecular-weight chitosan	- The microcapsules presented uniform particle size and encapsulation efficiency greater than 70%. - Particle size: 1–3 μm. - Oil stability has been improved by microencapsulation along with antibacterial antioxidant activity. - The release of CO from microcapsules revealed a rapid rate during the initial 5 h and a subsequent delayed release for up to 17 h. - Shelf life: 4 months at 25 °C.	[125]
**Nanoencapsulation**				
Nanoliposomes	Lentis essential oil (*Pistacia lentiscus* L.)	Soy lecithin	- Nanovesicles were considered ideal for treating skin wounds. - Nanovesicles promote the accumulation of bioactive in the dermis, neutralizing damage induced by oxidative processes. - Particle size: 118 nm.	[126]
	Chrysanthemum essential oil (*Chrysanthemum morifolium*)	Soy lecithin, chitosan, and pectin	- Single-layer liposomes (soy lecithin), double-layer liposomes (chitosan layer), triple-layer liposomes (chitosan-pectin layer) were prepared. - Triple-layer liposomes were more stable than other types (*p* < 0.05) and had high antibacterial activity against *Campylobacter jejuni* in chicken during 14 days of storage (4–37 °C), with no impact on chicken quality. - Particle size: 132.4 nm to 2148.4 nm.	[127]
	Barije essential oil (*Ferula gummosa*) (BEO)	Soy lecithin/cholesterol	- The nanoliposomal system containing BEO showed greater antimicrobial activity against *Escherichia coli* O157:H7 than the free form of BEO. - Particle size: 74.27 to 99.93 nm. - There was a gradual release of EO from the liposomes, which continued throughout the 24 h after inoculation.	[57]
	Clove essential oil (*Syzygium aromaticum* L.)	Saturated and unsaturated soy phospholipids/cholesterol	- Liposomes protected eugenol from degradation induced by UV exposure and maintained its elimination activity by DPPH. - Liposome formulations demonstrated stability after 2 months of storage at 4 °C.	[128]
Nanofibers	Mint essential oil (*Mentha piperita*) (MEO)	Poly (lactic acid)/polyethylene glycol (PLA/PEG)	- All nanofibers showed high thermal stability (278–345 °C). - Nanofibers with 20% MEO extended the shelf life of strawberries at 25 °C, showing the release of oil over time. - Particle size: 139–192 nm.	[129]
	Angelica essential oil (*Angelica sinensis* (Oliv.))	Gelatin	- The microcapsules showed an inhibitory effect against *E. coli* and *S. aureus* in a manner dependent on the gelatin concentration. - Particle size: 330.50 to 377.38 nm.	[54]
	Thyme essential oil (*Thymus vulgaris* L.)	Starch (50% *w*/*v*) and formic acid (75% *v*/*v*)	- Starch nanofibers showed high encapsulation efficiency (99.1% to 99.8%). - Free oil showed initial degradation from 62.1 °C while encapsulated oil started at 269.2 °C. - Particle size: 87.4 to 117.7 nm.	[130]
Molecular inclusion	Cymbopogon essential oil (*Cymbopogon martinii*)	β-cyclodextrin (CD)	- The inclusion complexes provided greater stability and bioavailability of the oil during storage. - The complexes showed better antifungal activity against *A. flavus* and *F. verticillioides.* - The complexes showed better activity against HT-29 cells when compared to HeLa cells. Free β-CD did not show antitumor activity in the assays.	[131]
	Wampee essential oil (*Clausena lansium*)	β-cyclodextrin	- The water solubility of the oil was clearly increased by 14 times after complexation. - Inclusion complexes preserved the antioxidant activity of the oil and improved its thermal stability.	[132]
	Nutmeg essential oil (*Myristica fragrans Houtt*.)	2-hydroxypropyl-β-cyclodextrin	- The ideal condition was inclusion temperature of 36 °C, time of 247 min, stirring speed of 520 r/min, and wall-to-core ratio of 12:1, resulting in a recovery of 80.63%. - The release of oil from the inclusion complex was controlled by regulating temperature and humidity. - There was an improvement in the thermal stability, antioxidant activities, and nitrite elimination of the oil after encapsulation.	[133]

**Table 3:** Studies on incorporation of encapsulated antimicrobials in different dairy products.

Product	Microbes	Natural Antimicrobial	Encapsulating Material	Outcomes	References
Milk	*Listeria monocytogenes*, *Salmonella enteritidis*, *Staphylococcus aureus*, and *Escherichia coli*	Nisin and garlic	Liposomes	A 1–4 log CFU/mL microbial load difference was observed between free and encapsulated nisin-GE.	[21]
	*Escherichia coli* and *Listeria monocytogenes*	Eugenol	Whey protein isolate and maltodextrin	Free and nanodispersed eugenol demonstrated the same antimicrobial characteristics, being more effective against Gram-negative *Escherichia coli* than Gram-positive *Listeria monocytogenes.*	[20]
	*Listeria monocytogenes*	*Thymus vulgaris* (Thymol)	Sodium caseinate	In skim milk, encapsulated thymol has slightly better antilisterial activity (ca. 1 log CFU/mL) than free thymol in the first 48 h, while no difference was observed at 72 or 168 h. The inactivation of *L. monocytogenes* was slower at a higher fat level and the encapsulated thymol consistently reduced the *L. monocytogenes* population to a lower level in a shorter time than free thymol. With 1.14% and 1.33% fat, both free and encapsulated thymol reduced *L. monocytogenes* to below the detection limit of 1 log CFU/mL in 168 h. With 1.49% fat, *L. monocytogenes* was reduced to about 2.5 log CFU/mL in 168 h by free and.	[157]
	*Staphylococcus aureus*, *Bacillus subtilis*, *Listeria monocytogenes* and *Listeria innocua*	*Schinus terebinthifolia* (Pink pepper)	Soy protein isolate and high methoxyl pectin	Reduction in the population below the detection limits of *S. aureus* and *L. monocytogenes*, between 4 and 6 Log CFU/mL. For *L. monocytogenes*, there was a reduction in the bacterial population of 2 Log CFU/mL.	[158]
Cheese	Total coliforms and *Staphylococcus*	*Cymbopogon Citratus* (lemongrass)	Arabic gum and maltodextrin	Microencapsulated oil reduced the growth count of coliforms at 45 °C and *Staphylococcus aureus*, corroborating with an increase in shelf life of 21 days.	[159]
	Mesophilic bacteria	*Rosmarinus offcinalis* (Rosemary)	Whey protein isolate and inulin	Addition of 0.5% microencapsulated essential oil reduced the mesophilic bacterial count 1.36 log cycles after three days and 0.73 log cycles after 15 days of storage.	[160]
	*Fusarium* sp., *Penicillium* sp. and *Cladosporium* sp.	*Origanum vulgare* (Oregano)	Sunflower oil, surfactants, and deionized water	Oregano essential oil encapsulated in nanoemulsions showed antifungal activity against the growth of *Cladosporium* sp., *Fusarium* sp. and *Penicillium* sp. *Penicillium* sp. showed greater resistance to the antifungal effect of oregano essential oil than *Cladosporium* sp. and *Fusarium* sp.	[161]
	*Filamentous* fungi and yeast	*Origanum vulgare* (Oregano)	Whey protein isolate	Microencapsulated oregano oil was effective in inhibiting the growth of fungi and yeast during 45 days of storage of grated cheese. Only the treatment containing 0.5% microencapsulated oil still had an undetectable count, being considered the most effective treatment in controlling filamentous fungi and yeast growth in grated Parmesan cheese.	[162]
	Aerobic mesophilic bacteria, molds and yeasts, and total coliforms	*Opuntia oligacantha* (Xoconostle)	Microcapsules: maltodextrin and gum arabic; nanoemulsion: soy lecithin and orange essential oil	Total coliforms decreased in all samples from the first days of storage (Control: 4.23 ± 0.12, Micro: 3.27 ± 0.02, and Nano: 2.68 ± 0.08 Log_10_ CFU), as well as aerobic mesophiles and mold–yeast counts.	[3]
	*Staphylococcus aureus,* Psychrophilic bacteria, molds and yeast	*Origanum vulgare* (Oregano)	Tween 80, sodium alginate, and mandarin fiber	The microbial population decreased by 1.4 and 1.5 log CFU/g in coated cheese pieces containing 2.0% or 2.5% *w*/*w* of oregano, respectively, during 15 days of refrigerated storage. However, coatings with a oregano concentration of 1.5% *w*/*w* were not effective in reducing *Staphylococcus aureus* population. Coated-cheese pieces containing 2.5% (*w*/*w*) oregano inhibited psychrophilic bacteria or molds and yeasts growth during 6 or 24 days of storage, respectively. However, a concentration of 1.5% *w*/*w* of oregano was not enough to inhibit the development of neither psychrophilic bacteria nor molds and yeast in cheese pieces.	[163]
Yogurt	*Escherichia coli* and *Staphylococcus aureus*	*Nepeta crispa*	Pectin, whey protein concentrate	The decrease in *Escherichia coli* and *Staphylococcus aureus* bacteria at 40 and 60 days of storage.	[1]
	*Escherichia coli* and *Staphylococcus aureus*	Garlic	composed of soy phosphatidylcholine	The inhibitory effect *Escherichia coli* than *Staphylococcus aureus* (minimum inhibitory concentration = 3.75 and 7.5 mg/mL, respectively).	[164]

## Data Availability

No new data were created or analyzed in this study. Data sharing is not applicable to this article.
